# Monthly Summary

**Published:** 1893-03

**Authors:** 


### Monthly Summary.

GOLD ATTACHMENTS IN CASES OF CLOSE BITE.—By W. A. Robertson, L. D. S., Crookston, Minn.—It frequently happens that we are called upon to replace one or more teeth by means of a plate, in which the bite is so close that it is not possible to use rubber as an attachment. So in cases when the patient can afford an all-gold plate, we are in the habit of attaching the teeth by an extension of gold plate into the rubber. Although this is nothing new, we have met several professional friends to whom it was new, and as it has been exceedingly useful to ourselves, as well as many others, we trust a short description of the process may not be amiss, and may be of use to others.

Having obtained a correct impression and bite, we select a plate tooth to correspond with the natural ones remaining, and grind it to fit closely to the gum. When this has been done, attach the tooth by a little wax to hold it in position, and varnish and oil the labial portion of the cast around the tooth, and run a little soft plaster over it, sufficiently to just cover the cutting edge, by using a little sulphate of potash. This only takes a minute or two, and is of great convenience in attaching the backing, etc. When hard remove the wax and investment, and back up the tooth in the ordinary way. We have found the use of a little fine cardboard (that in which the manufacturers send out the solder is generally handy) very convenient in shaping the backing. Press the pins through it and trim with a pair of scissors to the size the packing is to be, and by using this as a pattern, it is easy to cut the packing and punch the holes. When this is done, place the tooth back in the investment, and set back on the cast to see that the backing does not interfere with its going to place. If it is all right, cut a strip of gold plate (No. 30 is strong enough) about the same width for single teeth as the backing, and about one-half inch in length. Punch a few holes in this and bind to conform approximately to the roof of the mouth on the cast; lay it in place and close the bite to be sure it is right. Fasten to the backing with a little sticky wax, and remove from cast and invest in plaster and sand, equal parts, and solder. If the work has been carefully done, the soldered piece will go right to place, and the waxing up may be proceeded with. It is well to finish up the solder, etc., before waxing, as it is more troublesome to do when the plate is completed. When there are two or more teeth, we generally use the gold extension a little wider than for single teeth, attaching it midway between the two. This will be strong enough and saves time. In packing the rubber, draw out a small piece and then work it carefully under the gold extension, so as to insure it perfect imbedding in the rubber.—*Dominion Dental Journal.*

---

WATER AN ANÆSTHETIC.—The anæsthetic effects of cold water is much discussed at the Austrian capital. Dr. K. L. Schleich has discovered that absolute local immunity from pain, even during protracted operations, can be obtained without resorting to general narcosis of the patient, so that a sufferer may remain perfectly conscious during the amputation of his hand or foot without undergoing the tortures so often inflicted on the battle-field or exposing himself to the danger of syncope, ever present in the operating-room. It appears that subcutaneous injections of a solution of sugar or salt, nay, even of simple cold distilled water, will produce exactly the same local anæsthetic effects as cocaine. This discovery has already borne the test of numerous experiments, and will now be tried in Vienna. The explanation of the phenomenon is simple: Local insensibility to pain is caused in the case of cocaine by purely chemical changes, while cold water acts mechanically by means of high pressure and low temperature. Under the influence of the high pressure and sudden lowering of temperature the blood and lymph are driven from the region operated upon to places where the pressure is less. The tissue is thus deprived of its supply of blood and temporary paralysis of the nerves results. One of the first medical authorities affirms that the importance of this discovery is all the more undoubted, seeing that if, in a given case, cold water alone should fail to produce the needful degree of insensibility, a weak and absolutely harmless solution of cocaine would prove certainly efficacious.

---

DEATH FROM ALVEOLAR ABSCESS.—Although fortunately of a rare occurrence, it is well to bear in mind the possibility of a fatal issue in cases of alveolar abscess. From time to time instances are recorded where pyæmia is caused by absorption of septic material from the socket, and the treatment adopted by Mr. W A. Lane, in a case reported in the *Lancet*, is worthy of notice. A child of four years had an abscess in connection with a second temporary molar, which was removed, and the cavity was thoroughly cleaned out under an anæsthetic. The abscess had previously opened into the mouth, but there was much diffused swelling over the left half of the lower jaw. The boy became jaundiced and suffered from recurring rigors for four days. It was then decided to make an attempt to stay the progress of the condition by excising any thrombosed veins and clearing out inflamed tissues. The external jugular vein, which contained purulent material, and the facial vein, with their thrombosed branches, and many inflamed lymphatic glands, were removed. The patient was better the next day, but ultimately died, and at the post-mortem examination, a number of abscesses were found in the lungs and liver, produced before the operation. —*British Journal of Dental Science.*

ROOT CANAL CLEANSER.—We have received from Messrs. West and Schwarz, of Vienna, a preparation for removing the decayed matter in the root canals of teeth. It is introduced by Dr. Emil Schreier, who states that it is composed of potassium and sodium, and that it acts upon the decayed matter in a root by decomposing it. It produces a high temperature during the process, and effervesces, transforming the contents of the root canal into a soapy mass, which is easily removed, at the same time it renders the canal proof against the cultivation of new germs. It is applied with a canal cleanser, and the operation is to be repeated until no further chemical action takes place. Dr. Schreier states that after this preparation has been applied to the canal it is immaterial whether any particles of matter remain, as they are rendered harmless. He cautions dentists against the too frequent use of the same cleanser, as the preparation acts upon the steel and is liable to make it brittle. Air decomposes the material, but the oxidation is confined to the surface only, he recommends that the cork shall be pushed down on to the preparation each time after using; the bottles are made without necks for the purpose.—*Dental Record.*

---

FIRST AND SECOND MOLARS.—My study of the comparative liability of the teeth to decay at the different periods of life, shows that the first molars are attacked in the first two or three years after eruption, much oftener than any other tooth. But in after years they are attacked less often than the second. If both the teeth are in a fair condition at the age of fifteen, the chances for the first are better than those of the second. Decay occurs on the surface of the second molar much oftener than in the same locality in the first, and is much more difficult to treat.—*Dr. G. V. Black.*

